# Biomarkers to predict the outcomes of surgical intervention for aortic dissection

**DOI:** 10.1186/s13019-024-03226-4

**Published:** 2025-02-05

**Authors:** Abanob G. Hanna, Fabian Jimenez Contreras, Omar M. Sharaf, Griffin P. Stinson, Philip J. Hess

**Affiliations:** Department of Surgery, Division of Cardiovascular Surgery, University of Florida Health, 1600 SW Archer Rd, P.O. Box 100287, Gainesville, FL 32601 USA

## Abstract

**Purpose:**

Aortic dissections and repairs are associated with high rates of mortality. The aim of this review is to summarize the literature concerning the prognostic ability of various preoperative biomarkers for patients undergoing surgical repair of the thoracic and abdominal aorta to elucidate whether these biomarkers could improve the selection of surgical candidates.

**Methods:**

Relevant biomarkers were selected if they had predictive value in inflammatory disease processes and/or cardiovascular disease. Full-text articles available in English on PubMed that related these biomarkers to the prognosis of aortic repair following aortic dissection were examined.

**Results:**

For patients who underwent repair for type A dissection, a preoperative elevated SII was associated with inferior 30-day survival (hazard ratio: 3.532, 95% confidence interval: 1.719–7.255, *p* = 0.001) and increased rates of adverse cardiovascular events. Elevated preoperative IL-6 and D-dimer levels were independently associated with single-organ dysfunction, multiorgan dysfunction, and death. The use of a combination of markers was a stronger predictor. The areas under the curve (AUCs) for the specified IL-6 and D-dimer cutoff values were 0.901 and 0.817, respectively, whereas the AUC reached 0.936 when IL-6 was combined with D-dimer. For patients who underwent thoracic endovascular aortic repair (TEVAR) for type B dissection, an elevated postoperative systemic immune-inflammation index (SII) was an independent risk factor for aorta-related complications, graft failure, and significantly inferior freedom from aortic-related mortality. A combination of elevated preoperative and postoperative SII values was again predictive of in-hospital adverse outcomes and follow-up complications, including endoleaks, branch artery stenosis, distal aortic expansion, aortic rupture, and death (*p* = 0.0016). An elevated preoperative neutrophil‒leukocyte ratio (NLR) is associated with an increased incidence of early postoperative adverse events and poor survival.

**Conclusions:**

Inflammatory markers seem to have predictive ability for postoperative outcomes after aortic repair in type A dissections. Further studies should compare these biomarkers to determine the best predictive marker for individual disease states and surgeries.

## Introduction

Aortic dissection is both deadly and common in the United States; recent studies have suggested that the mortality rate of type A aortic dissection is as high as 5.78% at 48 h [1]. These diseases are particularly detrimental to patients’ lives, and their surgical repair is one of the most technically challenging and lethal cardiovascular procedures. Surgical repair of acute DeBakey type I aortic dissection carries a mortality risk as high as 17–18% [2]. Aortic disease repair also incurs a greater risk of stroke than do all other cardiac procedures [3]. People with an older age and/or history of chronic obstructive pulmonary disease and coronary artery disease can have a prolonged hospital length of stay (greater than 8 days) after aortic disease repair [3]. Therefore, a better understanding of how to manage patients, especially those with comorbidities and risk factors, is warranted.

There is a preponderance of evidence that links aortic dissection to inflammation [4]. Although aortic dissection is considered a multifactorial disease, inflammatory mechanisms have been associated with weakening of the tunica medica. Histopathological evidence has demonstrated the increased presence of T lymphocytes and macrophages and elevated levels of apoptotic markers [4]. The serum levels of inflammatory markers might provide insight into the extent of the disease in patients [4]. In addition to histopathological indications of inflammation, increased plasma levels of inflammatory markers have been determined to be independent risk factors for various cardiovascular diseases [4]. The literature has increasingly shown how inflammatory processes and the development of aortic dissection are tightly connected. The question addressed in this literature review is whether patients in a more advanced stage of the disease from an inflammatory perspective might prove to have worse postoperative complications. In other words, the aim is to link inflammatory processes to postoperative outcomes by providing a summary of the current literature discussing the prognostic ability of various inflammatory markers in patients with aortic dissection.

## Methods

In a PubMed search, the following MeSH terms were used to identify relevant literature: “thoracic aortic dissection” and “abdominal aortic dissection.” The search was further restricted to “surgery” subheading for each MeSH term. Using PubMed Advanced Search Builder, “prognosis” and the name of a biomarker were combined in the query box with the listed MeSH terms via the “add with AND” function. Inflammatory biomarkers were selected if they had shown predictive value in other inflammatory disease processes and/or cardiovascular disease. The list of selected biomarkers is as follows: systematic immune inflammatory (SII), IL-6, D-dimer, procalcitonin (PCT), brain natriuretic peptide (BNP), C-reactive protein (CRP), matrix metalloproteinase-9 (MMP-9), homocysteine (HCY), and osteoprotegerin (OPG). This yielded an initial list of studies. After which, a citation analysis was conducted using PubMed, and new articles were included. Full-text articles available in English were eligible for inclusion. We included studies that investigated the relationship of any inflammatory biomarker with outcomes of type A or type B aortic dissection repair. This is regardless of the type of study, the sample size, the date of publication, and where the study was conducted. Studies were not included if they were not primarily focused on dissection repair.

It is of note that SII is a calculation that attempts to index the inflammatory state by multiplying peripheral blood platelets by the ratio of neutrophils to leukocytes. It has been clinically used with various malignancies.

## Results

### Type A dissection

In patients who underwent a repair procedure for type A dissection, preoperative systemic immune‒inflammation index (SII) measurements were positively correlated with both 30-day mortality (hazard ratio: 3.532, 95% confidence interval: 1.719–7.255, *p* = 0.001) and major adverse cardiovascular events (MACE), including myocardial infarction, malignant ventricular arrhythmia, hospitalization because of heart failure, and the use of a mechanical assist device [5]. In a retrospective study by Xu and colleagues, a cohort size of 324 patients was subdivided into a low SII group and a high SII group, with a cutoff SII value of $\:{1582.6}\times{\text{10}}^{\text{9}}$/L^4^. This cutoff value was defined by a receiver operating characteristic (ROC) curve. According to a Kaplan‒Meier analysis, 30-day mortality was greater in the high-SII subgroup than in the low-SII subgroup [5]. Additionally, in a multivariable analysis, including the SII, hypertension, acute renal failure, cardiopulmonary bypass time, and deep hypothermic circulatory arrest time, the SII was shown to be an independent risk factor for 30-day mortality [5]. Finally, the incidences of MACE and multiorgan failure were greater in the high-SII subgroup than in the low-SII subgroup [5]. Similar results were shown in a different study by Li and his colleagues [6]. In which, a group of 496 type A dissection patients were subdivided into high SII and low SII groups based on a cut-off value determined by a ROC plot [6]. A striking difference between the Xu and Li study is the cut-off value used for SII. In the Li study, the cut-off value is 3324.5 $\:\times{\text{10}}^{\text{9}}$/L^4^ twice the cut off of the Xu study. Patients in the high SII group had statistically significant higher short-term mortality (*p* < 0.001). In this study also, a Kaplan-Meier curve was also constructed which showed that the overall survival was higher in the low SII group (HR: 0.32; 95% CI: 0.21–0.49, *p* < 0.001) [6].

In another study on type A aortic dissection (TAAD) surgery patients, procalcitonin (PCT) levels were assessed for their prognostic value in terms of complications and surgical outcomes [7]. PCT levels peak 24 h post-surgery and are correlated with cardiopulmonary bypass duration. Higher PCT levels were observed in non-survivors and those with multiple organ dysfunction syndrome [7]. PCT at 24 h after surgery was linked to prolonged ventilation and ICU/hospital stays. A PCT level of 5.86 ng/ml at 48 h post-surgery had a sensitivity of 70.6% and specificity of 74.3% for the prediction of in-hospital death [6]. PCT clearance (PCTc) on days 2 and 7 was significantly greater in survivors, with a day 7 PCTc cutoff of 48.7% predicting survival, with 77.8% sensitivity and 81.8% specificity [7].

In addition to the SII and PCT, preoperative interleukin-6 (IL-6) has shown prognasitc value alone and when combined with other markers, such as D-dimer levels. In a small retrospective cohort study of 141 patients by Wu et al., IL-6 concentrations greater than 108 pg/mL and D-dimer concentrations greater than 14.0 µg/mL, including respiratory failure, liver failure, digestive system dysfunction, sepsis, and multiple organ dysfunction (listed in order of prevalence), predicted early poor postoperative prognosis [8]. Like the Xu et al. study, the IL-6 and D-dimer cutoff values were determined via ROC curves. The areas under the curve (AUCs) for the specified IL-6 and D-dimer cutoff values were 0.901 and 0.817, respectively, whereas the AUC reached 0.936 when IL-6 was combined with D-dimer [8]. Therefore, the combination of the two markers was determined to be a better predictor. A summation of preoperative and postoperative IL-6 greater than 83.4 pg/mL was also shown to have 91.5% sensitivity and 78.2% specificity in predicting postoperative prolonged mechanical ventilation lasting more than 48 h [9]. Pre-operative IL-6 also had 61.3% sensitivity and 79.4% specificity of patients developing post-operative delirium [10].

In a study by Xie and his colleagues, D-dimer demonstrated to be an independent risk factor for postoperative clinical adverse events in type A aortic dissection patients, including renal failure, respiratory failure, GI bleed, low cardiac output syndrome, ventricular fibrillation, permanent neurological deficits, sepsis, death etc [11]. The AUC for a ROC plot was 0.77. Interestingly, D-dimer combined with prognostic nutritional index (PNI) also had a better predictive value (AUC = 0.84) [11]. An additional study was conducted to examine the value of pre-operative D-dimer, C-reactive protein (CRP), and matrix metalloproteinase 9 (MMP-9) by Huang and his colleagues [12]. In this study, 247 patients were included. It was found that among the deceased group (death within 1 year after surgery), subjects had higher CRP, D-dimer, and MMP-9 [12]. A combined detection model using preoperative blood CRP, D-dimer, and serum MMP-9 concentrations showed AUC value of 0.88 with 70.70% sensitivity and 96.84% specificity for mortality of patients with Type A aortic dissection within 1 year [12].

Other novel inflammatory markers were also examined to determine their prognostic value in patients with Type A aortic dissection, such as S100A8/A9, pentraxin 3, chitinase 3-like 1, and S100B. In a study by Wang and his colleagues, including 328 patients, ROC curves were constructed to predict surgery associated acute kidney injury [13]. AUC values for S100A8/A9, PTX3, and CHI3L1 at 0 h post-surgery were 0.823, 0.786, and 0.803, respectively [13]. In a meta-analysis of 8 studies conducted by Si, S100B were shown to have value in predicting post-operative neurological complications [14]. In patients with moderate or severe brain injury, S100B concentrations continue to increase, reaching an upper limit at 24 h after cardiopulmonary bypass [14]. On the other hand, in patients with no or low brain injury; S100B concentrations peaked at 6 h and declined [14].

In another study by Zhao and his colleagues, a model that combines several blood markers including leukocyte, neutrophils, monocyte, lymphocytes, platelet, fibrinogen, d-dimer, NLR, platelet-lymphocyte ratio (PLR), and lymphocyte-monocyte ratio (LMR), was constructed to separate patients into a hyper-inflammatory group and a hypo-inflammatory group [15]. A multivariate analysis showed that patients in the hyper-inflammatory profile had 2.699 the odds of death in 30 days and 2.427 the odds of death in 6 months [15].

### Type B dissection

Other investigators focused on patients with type B dissection who underwent thoracic endovascular aortic repair (TEVAR) procedures. Zhao’s retrospective study of 186 participants revealed that the postoperative neutrophil‒lymphocyte (NLR) ratio and the SII were both elevated in the group that developed aorta-related adverse events (AAEs), such as distal abdominal aortic expansions, endoleaks, distal stent-induced new entries, retrograde type A dissection (RTAD), and death due to aortic rupture ($\:p=0.023$ and $\:p=0.016$, respectively) [16]. The AUC for a postoperative SII greater than 2893 was 0.520 [16]. According to the multivariate logistic regression analysis, both an SII greater than 2893 and age were found to be independent risk factors for AAE following TEVAR. Kaplan‒Meier AAE-free survival curves revealed that patients whose postoperative SII was greater than 2893 had a lower median follow-up period and survival. The author noted that high SII values were due to increased platelet counts [16]. In a later and larger retrospective study of 806 participants by Su et al., the ROC value for the baseline preoperative SII was 0.60 at a cutoff value of $\:\text{1062}{\times}{\text{10}}^{\text{6}}$/L [18]. In-hospital major adverse cardiovascular events (MACEs) in the high-SII group were almost one-time higher than those in the low-SII group, and mortality rates were almost twofold higher [17]. Multivariate logistic analyses revealed that a preoperative SII>$\:\text{1062}{\times}{\text{10}}^{\text{6}}$/L was an independent predictor of MACEs [9]. Both studies by Zhao and Su showed that both preoperative and postoperative SII values can have clinical value in predicting the prognosis of patients with acute type B dissection following TEVAR.

Zhu and colleagues focused on the neutrophil-to-lymphocyte ratio (NLR) instead of the SII (which is derived from multiplying the NLR by the platelet count) [18]. They investigated the preoperative NLR values of patients who experienced acute onset of uncomplicated type B dissection, successful TEVAR, and no in-hospital adverse events. The participants were divided into a low NLR group and a high NLR group, with a cutoff value of 4.8, which was determined by an ROC curve [15]. Multivariate regression analysis revealed that a high preoperative NLR was an independent factor for poor 2-year overall adverse event-free survival [18]. The observed adverse effects were distal abdominal aortic expansion, endoleaks, distal stent-induced new entry, retrograde type A dissection (RTAD), and aortic rupture. NLR was also found to be an independent risk factor for in-hospital death by Yang and his colleagues in a multivariate study including 841 patients [19]. Importantly, the Yang study had a very similar cut-off value as the Zhu study of 0.41 [19].

In a unique study of 912 patients with type B aortic dissection which was conducted by Zhao and his colleagues, Multivariable regression analysis showed association between eosinophils count and long-term outcomes [20]. Lower eosinophils count was associated with significantly higher 30-day mortality, 1-year all-cause mortality, and aortic related death [20]. Eosinophils count was shown to be an independent risk factor for 1-year mortality [20]. CD10 − immature neutrophils was another granulocyte that had shown some predictive ability in type B aortic dissection patients as shown in a study by Bokha [21]. Precent immature neutrophils was 30.82% in the group of patients that developed aortic adverse events vs. 28.54% in the event-free group [21]. Following multivariate analysis, type B aortic dissection patients with higher percentage of immature neutrophils had higher aortic adverse event rate than those with lower percent immature neutrophils (HR: 7.66, 95% CI: 2.91, 20.17, *P* = 0.018) [21] (Table 1).

**Table 1:** Summary table of the studies investigated in this literature review

Study ID	Period of enrollment	Design (number of subjects/studies)	Prognostic biomarkers	Outcomes	Type of analysis
35141947	2018–2020	Retrospective study (324 Type A aortic dissection patients)	SII	**30-day mortality** (HR: 3.532, 95% CI: 1.719–7.255, *p* = 0.001) Higher postoperative complications including **major adverse cardiovascular events** (*p* = 0.001) and **multiorgan failure** (*p* = 0.002)	Multivariable analysis
36238764	2016–2018	Retrospective study (496 Type A aortic dissection patients)	SII	**In-hospital death** (OR: 4.116; 95% CI: 1.13–2.47, *p* < 0.001) **Postoperative survival** (HR: 2.467; 95% CI: 1.573–3.868, *p* < 0.001	Multivariable analysis
28485317	2013–2015	Retrospective study (251 Type A aortic dissection patients)	PCT	Correlation existed between PCT levels at T1 and **duration of CPB** (*P* = 0.001, *r* = 0.278) Correlation existed between serum PCT concentration at Time 1 and **the duration of endotracheal intubation** (*P* < 0.001, *r* = 0.390), duration of stay in the ICU (*P* < 0.001, *r* = 0.371), and **duration of stay in the hospital** (*P* = 0.008, *r* = 0.182) Serum PCT level > 6 ng/ml at time 2 (OR = 7.473, 95% confidence interval [CI] = 1.061–32.885, *P* = 0.043) were associated with **30-day mortality**	Multivariable analysis
32677975	01/2018–12/2018	Retrospective study (141 Type A aortic dissection patients)	IL-6, D-dimer,	IL-6 > 108 pg/mL and D-dimer > 14.0 µg/mL **predicted postoperative risk factors for poor prognosis** (OR: 24.937 and 18.757 respectively) Sensitivity and specificity of IL-6 > 108 pg/mL of predicting **poor early postoperative prognosis** are 79.4% and 89.7%, respectively (95% CI 0.839 to 0.963) Sensisitivity and specificity of D-dimer > 14 g/mL of predicting **early postoperative prognosis** are 82.4% and 84.1% respectively (95% CI 0.731 to 0.903) When combined with D-dimer (AUC = 0.936) (95% CI 0.793 to 0.979), the AUC values were more predictive of **early poor postoperative prognosis** than those for the individual marker.	Multivariable analysis
37123919	06/2018–06/2022	Retrospective study (381 Type A aotic dissection patients)	IL-6, D-dimer	IL-6 (post-IL-6) ≥ 67.1 pg/mL and summation of preoperative and postoperative IL-6 (total IL-6) ≥ 83.4 pg/mL were associated with **PMV** [(OR: 3.259, CI: 1.922–5.524, *p* < 0.001], [(OR): 4.515, 95% CI 2.241–9.098] Sensitivity and specificity of predicting postoperative **PMV** for total IL-6 ≥ 83.4 pg/mL were 91.5% and 78.2%, respectively (95% CI 0.782–0.868, *p* < 0.001),	Multivariable analysis
37876775	01/2019–01/2022	Retrospective study (153 Type A aortic dissection patients)	D-dimer, PNI	OR of **clinical adverse events** for preoperative PNI < 42.45 is 3.596 [95% CI: 1.508–8.923, *p* = 0.004], OR of **clinical adverse events** for D-dimer > 15.05 is 7.572 [95% CI: 3.094–20.220, *p* < 0.001]. The combination of PNI and D-dimer has a high predictive value for **clinical adverse events** (AUC = 0.843, 95% CI: 0.774–0.912, *p* < 0.001)	multivariate analysis
37849442	01/2018–01/2022	Retrospective study (247 Type A aortic dissection patients)	D-dimer, CRP, MMP-9	AUC of preoperative CRP level predicting **the 1-year survival** in patients with ATAAD was 0.69 [95% CI: 0.62 to 0.77, sensitivity 50.56%, specificity 86.71%, *P* < 0.001]. The AUC of preoperative d-dimer level predicting **the 1-year survival** was 0.82 (95% CI: 0.76 to 0.88, sensitivity 67.42%, specificity 87.97%, *P* < 0.001). The AUC of preoperative MMP9 level predicting **the 1-year survival** in patients with ATAAD was 0.74 (95% CI: 0.67 to 0.81, sensitivity 62.92%, specificity 79.11%, *P* < 0.001). The AUC of a joint prediction model combining D-dimer, CRP, MMP-9 was 0.88 (95% CI: 0.84 to 0.94, sensitivity 70.79%, specificity 96.84%, *P* < 0.001).	multivariate analysis
38110934	06/2021–03/2023	Prospective study (328 Type A aortic dissection patients)	S100A8/A9, Pentraxin 3, Chitinase 3-like 1	AUC of S100A8/A9, PTX3, and CHI3L1 at 0 h post-operatively in predicting **AKI** are 0.823 (CI: 0.723–0.926), 0.786 (CI: 0.679–0.893), and 0.803 (CI:0.700-0.905) respectively. OR of **AKI** for elevated S100A8/A9 at 0 h for: 1.33 (CI: 1.18–1.54, *p* < 0.001) OR for **AKI** of elevated PXT3 at 0 h PTX3: 1.25 (CI: 1.13–1.41, *p* < 0.001) OR of **AKI** for elevated CHI3L1 at 0 h: 1.01 (CI: 1.00-1.01, *p* < 0.001)	Multivariate analysis
36753509	Up to 02/2022	Meta-analysis (12 studies – 1126 Type A aortic dissection patients)	S100B	S100B levels at 6 h post-operatively were associated with the **increased risk of neurological complications** (WMD = 0.64, 95% CI: 0.27 ~ 1.02, *P* = 0.0007 < 0.001, I^2^ = 97%) S100B levels at 24 h post-operatively associated with the increased risk of **neurological complications** (WMD = 0.26, 95% CI: 0.12 ~ 0.39, *P* = 0.0002 < 0.001, I^2^ = 94%)	
37713048	01/2018–12/2020	Prospective study (193 patients)	leukocyte, neutrophils, monocyte, lymphocyte, platelet, fibrinogen, d-dimer, NLR, PLR, LMR	hyper-inflammatory profile was associated with higher 30-day mortality (OR: 2.699, 95% CI: 1.201–6.064, *P* = 0.016) and 6-month mortality (OR: 2.427, CI: 1.154–5.105, *P* = 0.019) than hypo-inflammatory profile.	Multivariate analysis
36845014	11/2016–11/2020	Retrospective study (186 Type B aortic dissection patients)	NLR, SII	The postoperative NLR and SII were different between the (**aortic adverse event) AAE** and non-AAE groups (*p* = 0.023 and *p* = 0.016, respectively). AUC of post-operative SII greater than 289 and NLR greater than 6.703 for predicting **AAE** is 0.5204 and 0.5841 respectively OR of **AAE** for post-operative SII is 2.48 (CI: 1.02–6.14, *p* = 0.04) HR of **AAE** for post-operative SII is 1.88 (CI: 1.02–3.48, *p* = 0.043)	Multivariable analysis
34695253	2010–2017	Prospectively maintained database (805 patients with Type B aortic dissection patients)	SII	SII greater than 1,062 was associated with **major adverse cardiovascular events (MACEs)** in-hospital (OR: 1.67; CI: 1.13–2.47; *p* = 0.01). SII greater than 1,062 was associated with **follow-up adverse events** (HR: 1.70; 95% CI, 1.14–2.56, *p* = 0.01).	Multivariate analysis
33593284	01/2015–01/2018	Retrospective study (216 Type B aortic dissection patients)	NLR	Pre-operative NLR had HR of 1.98 (CI:1.14–3.44; *P* = 0.015) on **2-year overall event-free survival**	Multivariate analysis
33955277	2010–2017	Retrospective study (841 type B aortic dissection patients)	NLR	NLR predicted in-hospital death (OR: 1.15; CI: 1.09–1.22; *P* < 0.001)	Multivariate analysis
36416154	01/2003–07/2019	Retrospective study (912 type B aortic dissection patients)	Eosinophils count	eosinophils < 7.4 × 10^6^/L had a higher incidence of 1-year all‐cause death (7.95% vs. 2.34%, *P* = 0.008) and aortic‐related death (5.98% vs. 1.81%, *P* = 0.023) continuous eosinophils was independently associated with 1-year mortality (HR: 3.23, CI: 1.20–8.33, *P* = 0.019).	Multivariate analysis
37637713	01/2019–03/2021	Retrospective study (80 type B aortic dissection patients)	CD10 − immature neutrophils	higher % of immature neutrophils had a higher aortic adverse events rate than those with lower percent immature neutrophils (HR: 7.66, 95% CI: 2.91, 20.17, *P* = 0.018)	Multivariate analysis

## Discussion

In this literature review, we attempted to summarize the literature on the prognostic abilities of various inflammatory markers in aortic dissection patients. A total of 16 studies were included. Six studies examined patients with type B aortic dissection, and the rest examined patients with type A aortic dissection. Most of the studies were retrospective observational analysis in exception to 3 prospective studies and 1 meta-analysis. The earliest study included patients from 2010, and the latest enrolled patients until 2023. The smallest study included 80 subjects while the largest study was meta-analysis which included 1126 subjects. Invariably, all the studies conducted a univariate analysis to determine factors that have statistically significant effects on outcomes followed by a multivariate analysis of these specific factors.

Inflammation has been shown to be an integral part of the development of dissection. The sprouting research attempts to use this knowledge in a clinical setting. One such way is to use the inflammatory state of patients as indicated by several inflammatory markers as predictors of the patients’ prognosis. The literature, overwhelmingly, shows correlation between a worse inflammatory state and a worse post-operative prognosis in both type A and type B aortic dissection patients.

Two markers sparked special interest - the neutrophil-to-leukocyte ratio (NLR) and the systemic immune-inflammation index (SII) which is the NLR multiplied by the platelet count. Both of which represent a composite of a patient’s inflammatory state and are easily calculated and commonly collected in patients undergoing repair of a dissection. Across three studies with multivariate analysis, patients with SII were shown to have higher odds of aortic-adverse events, in-hospital death, and 30-day mortality in type A aortic dissection patients. Type B aortic dissection patients had higher odds of major adverse cardiovascular events (MACE) and follow-up adverse events. High neutrophil-to-leukocyte ratio (NLR) was also shown to increase the odds of in-hospital death.

Literature shows similarity between type A and type B aortic dissection in that the prognosis of their repair is associated with the inflammatory state. Moreover, as discussed in the earlier paragraph, SII seems to be valuable in both processes. There is not a clear agreement on a cut-off value for SII to separate patients with bad prognosis from good prognosis. Though, two studies had agreed on a similar cut-off value which is around $\:\text{3000}$; one predicted short-term mortality in type A aortic dissection patients, and the other predicted aortic-related adverse events in type B aortic dissection. Besides SII, higher D-dimer values were demonstrated to be predictive of worse prognosis in Type A dissection patients especially when combined with various other biomarkers. In type B aortic dissection, higher eosinophils and CD10 − immature neutrophils counts were associated with higher mortality and aortic adverse events respectively.

Another significant take-away from literature is that often inflammatory markers alone show lower sensitivity and specificity. However, models that combine these markers have significantly higher predictive value. One example of this is one of the models that combined D-dimer, CRP, and MMP-9 to predict 1-year mortality in type A aortic dissection patients. Preoperative CRP level alone had sensitivity of 50.56% and specificity of 86.71%. D-dimer level had sensitivity of 67.42% and specificity of 87.97. Preoperative MMP9 level had sensitivity 62.92% and specificity of 79.11%. However, A joint prediction model combining D-dimer, CRP, MMP-9 had sensitivity 70.79% and specificity 96.84%. In other words, these markers are best used as part of a larger clinical picture.

It is of note that many of the markers examined in the literature and included in this review are not commonly collected as standard of care. This includes but not limited to IL-6, MMP-9, CRP, S100B, S100A8/A9, Pentraxin 3, and Chitinase 3-like 1. However, expanding the range of routinely assessed markers to include these novel indicators could be a valuable endeavor as they demonstrate prognostic value as shown in several of the studies in this review. By casting a wider net in the search for meaningful prognostic data, it may be possible to identify markers that, if validated through further research, could significantly enhance patient care and outcomes by providing more precise and comprehensive prognostic information as either stand-alone markers or as components of predictive models.

As shown in this literature review, current research is rather promising. However, there is a growing need for a large multi-center study based in the United States to confirm the existent data and elaborate on the clinical usefulness of these markers.

## Conclusion

There is evidence in support of a positive correlation between elevated levels of inflammatory markers and worse prognosis after repair of aortic dissection. While these markers individually are not highly specific or sensitive for a worse prognosis, they have been repeatedly proven to be independent risk factors for worse prognosis and particularly useful when combined in a predictive model. Therefore, they have the potential to be tools in the hands of surgeons to improve their decision-making process when surgical repair is not an obvious decision. However, a definitive assessment of their clinical value has not yet been performed. This is because there is not a large body of research on this topic. Moreover, many of the current studies, as shown in this literature review, are institutionally based retrospective studies with small sample sizes. Larger clinical studies, especially those assessing the prognostic ability of the systemic immune-inflammation index (SII), are needed not only to better understand the correlation between inflammatory markers and postoperative outcomes but also to develop useful clinical recommendations.

## Data Availability

No datasets were generated or analysed during the current study.

## Abbreviations

AUC: Area under the curve
TEVAR: Thoracic Endovascular Aortic Repair
SII: Systemic immune-inflammation index
CI: Confidence interval
NLR: Neutrophil-leukocyte ratio
PLR: Platelet-lymphocyte ratio
LMR: Lymphocyte-monocyte ratio
PCT: Procalcitonin
PCTc: Procalcitonin clearance
PNI: Prognostic nutritional index
BNP: Brain natriuretic peptide
CRP: C-reactive protein
MMP-9: Matrix metalloproteinase-9
HCY: Homocysteine
OPG: Osteoptrotegrin
MACE: Major adverse cardiovascular event
ROC: Receiver operating characteristic
TAAD: Type A aortic dissection
IL-6: Interleukin-6
AAE: Aorta-related adverse events
RTAD: Retrograde type A dissection
OR: Odds ratio
HR: Hazard ratio
CI: Confidence interval
